# Supplementary material for: Post-Radiotherapy Exosomal Non-Coding RNA and Hemograms for Early Death Prediction in Patients with Cervical Cancer
Source: Int J Mol Sci. 2023 Dec 21;25(1):126. doi: 10.3390/ijms25010126 (PMC10778718; doi:10.3390/ijms25010126)
Supplement: Supplementary file 1 [file ijms-25-00126-s001.zip › supplementay_tables_and_figures.pdf]

Table S1. Comparison results of hematological parameters among groups 1 (early death), 2 (progression), and 3 (non-progression) acquired through propensity score matching (Cohort 1)

	Group1 (N=24)	Group2 (N=24)	p	Group2 (N=48)	Group3 (N=48)	p	Group1 (N=24)	Group3 (N=24)	p
Age	55.04 ± 14.23	55.88 ± 11.06	0.822	54.96 ± 13.07	52.52 ± 11.13	0.328	55.04 ± 14.23	52.50 ± 11.28	0.496
pathology			1			1			1
sqcc	21 (87.50%)	21 (87.50%)		43 (89.58%)	43 (89.58%)		21 (87.50%)	21 (87.50%)	
non-sqcc	3 (12.50%)	3 (12.50%)		5 (10.42%)	5 (10.42%)		3 (12.50%)	3 (12.50%)	
FIGO stage			1			1			1
2A-3C1	15 (62.50%)	15 (62.50%)		39 (81.25%)	39 (81.25%)		15 (62.50%)	15 (62.50%)	
3C2-4A	9 (37.50%)	9 (37.50%)		9 (18.75%)	9 (18.75%)		9 (37.50%)	9 (37.50%)	
RT_field			1			1			1
pelvis	14 (58.33%)	14 (58.33%)		32 (66.67%)	32 (66.67%)		14 (58.33%)	14 (58.33%)	
pelvis c PA	10 (41.67%)	10 (41.67%)		16 (33.33%)	16 (33.33%)		10 (41.67%)	10 (41.67%)	
TD(EQD2)	71.62 ± 6.91	73.97 ± 3.88	0.155	72.25 [69.04;75.88]	72.25 [68.99;74.23]	0.686	71.36 [67.58;75.36]	72.25 [69.28;72.81]	0.468
OT	59.00 ± 9.53	56.33 ± 9.92	0.347	52.00 [49.50;60.00]	56.00 [51.00;63.00]	0.087	59.00 ± 9.53	54.38 ± 5.52	0.047
SCC0	12.30 [1.95;27.95]	5.05 [1.60;13.50]	0.271	8.50 [1.70;30.60]	4.15 [1.60;13.90]	0.125	12.30 [1.95;27.95]	9.40 [2.00;21.60]	0.809
SCC2	1.80 [1.00;3.80]	1.30 [0.50;4.30]	0.391	2.40 [0.60;7.70]	1.00 [0.80;2.60]	0.262	1.80 [1.00;3.80]	1.05 [0.80;2.05]	0.14
Cyfra0	5.25 [2.15;10.05]	6.40 [2.50;12.70]	0.422	5.80 [2.60;11.00]	2.25 [1.50;4.20]	0.001	5.25 [2.15;10.05]	2.25 [1.50;19.00]	0.286
Cyfra2	1.80 [1.20;2.55]	1.80 [1.20;3.60]	0.934	1.80 [1.20;3.45]	1.60 [1.00;2.65]	0.165	1.80 [1.20;2.55]	1.55 [1.00;3.00]	0.677
Hb0	10.80 ± 1.65	10.94 ± 1.60	0.77	11.05 [9.65;12.10]	12.15 [10.75;13.15]	0.008	10.80 ± 1.65	11.60 ± 1.56	0.093

Min Hb	9.10 ± 1.29	9.12 ± 0.82	0.958	9.00 [8.50;9.85]	9.65 [8.90;10.60]	0.073	9.10 ± 1.29	9.27 ± 1.57	0.698
Hb1	9.90 ± 1.32	9.97 ± 1.04	0.834	9.94 [9.10;10.91]	10.59 [9.79;11.74]	0.016	9.90 ± 1.32	10.35 ± 1.48	0.272
Hb3	-0.04 ± 0.14	-0.01 ± 0.13	0.502	-0.04 [-0.11;0.04]	-0.06 [-0.10;-0.01]	0.156	-0.05 [-0.12;0.04]	-0.05 [-0.08;-0.01]	0.959
PLT0	305.50 [247.50;343.50]	268.50 [240.50;349.50]	0.503	261.00 [222.00;297.00]	280.00 [249.50;320.00]	0.206	305.50 [247.50;343.50]	271.50 [215.50;300.50]	0.122
Min PLT	148.96 ± 52.01	126.33 ± 46.88	0.12	131.65 ± 45.50	134.60 ± 36.67	0.727	148.96 ± 52.01	117.96 ± 33.35	0.018
PLT1	209.60 ± 51.99	187.55 ± 52.29	0.15	186.30 ± 51.40	194.32 ± 45.62	0.421	198.96 [171.16;245.80]	173.40 [144.30;199.01]	0.027
PLT3	-0.32 ± 0.26	-0.28 ± 0.29	0.605	-0.26 [-0.40;-0.11]	-0.27 [-0.48;-0.16]	0.228	-0.32 ± 0.26	-0.29 ± 0.19	0.634
Mo0	511.80 ± 233.11	444.25 ± 143.83	0.234	405.77 ± 171.70	495.68 ± 158.42	0.009	511.80 ± 233.11	522.81 ± 160.89	0.85
Min Mo	245.98 ± 118.34	196.15 ± 143.84	0.196	203.79 ± 131.53	250.65 ± 96.63	0.05	245.98 ± 118.34	245.83 ± 107.26	0.996
Mo1	339.53 ± 130.68	279.11 ± 144.28	0.135	271.14 ± 134.21	344.66 ± 106.07	0.004	339.53 ± 130.68	347.93 ± 111.22	0.811
Mo3	-0.30 [-0.63;0.17]	-0.10 [-0.37;0.10]	0.66	-0.12 [-0.38;0.11]	-0.22 [-0.37;-0.02]	0.344	-0.30 [-0.63;0.17]	-0.25 [-0.33;-0.06]	0.767
ANC0	5273.55 [4290.66;7362.00]	4624.85 [3527.04;5901.95]	0.048	4489.32 [3527.04;5726.22]	4356.81 [3750.65;6187.30]	0.547	5273.55 [4290.66;7362.00]	4271.40 [3765.00;6976.95]	0.141
Min ANC	2150.27 ± 1021.49	1492.79 ± 771.06	0.015	1800.23 ± 839.63	1685.38 ± 592.56	0.441	2150.27 ± 1021.49	1573.09 ± 646.96	0.025
ANC1	3290.47 [2810.28;3891.17]	2474.06 [1984.73;3111.41]	0.009	2843.89 ± 965.10	2853.86 ± 806.19	0.956	3290.47 [2810.28;3891.17]	2772.70 [1990.64;3432.66]	0.036
ANC3	-0.41 [-0.70;-0.13]	-0.33 [-0.63;-0.11]	0.533	-0.32 [-0.59;-0.11]	-0.26 [-0.56;0.00]	0.523	-0.41 ± 0.40	-0.25 ± 0.43	0.193
ALC0	1590.99 ± 575.39	1821.70 ± 424.55	0.121	1755.72 ± 578.58	1966.47 ± 644.65	0.095	1590.99 ± 575.39	1950.58 ± 647.66	0.048
Min ALC	193.45 [144.79;321.55]	177.12 [137.16;260.10]	0.31	220.37 [156.05;286.95]	268.15 [169.16;379.60]	0.115	235.41 ± 128.00	263.81 ± 120.47	0.433
ALC1	506.69 [396.25;724.11]	550.00 [453.78;650.96]	0.846	579.28 [469.12;781.90]	713.40 [506.60;944.61]	0.047	593.95 ± 249.14	703.91 ± 247.47	0.132

ALC3	-1.14 ± 0.41	-1.27 ± 0.51	0.329	-1.19 ± 0.46	-1.11 ± 0.40	0.377	-1.14 ± 0.41	-1.16 ± 0.43	0.862
NLR0	4.17 [2.84;5.97]	2.56 [1.78;3.37]	0.003	2.66 [1.81;3.60]	2.41 [1.73;3.65]	0.617	4.17 [2.84;5.97]	2.55 [1.77;3.81]	0.009
NLR1	6.33 [4.61;8.70]	4.42 [2.87;7.12]	0.022	4.53 [2.93;6.55]	4.01 [3.16;5.06]	0.202	6.33 [4.61;8.70]	4.07 [3.26;5.08]	0.002
PLR0	0.20 [0.14;0.30]	0.15 [0.12;0.21]	0.046	0.15 [0.12;0.19]	0.14 [0.12;0.19]	0.49	0.20 [0.14;0.30]	0.14 [0.11;0.18]	0.018
PLR1	0.35 [0.27;0.49]	0.32 [0.24;0.45]	0.31	0.28 [0.23;0.41]	0.27 [0.21;0.36]	0.42	0.35 [0.27;0.49]	0.26 [0.20;0.35]	0.011
LMR0	3.04 [2.19;5.27]	4.31 [3.25;4.97]	0.125	4.53 [3.35;5.82]	3.98 [3.10;5.18]	0.12	3.04 [2.19;5.27]	3.98 [2.79;4.98]	0.407
LMR1	1.75 [1.37;2.67]	2.43 [1.63;2.99]	0.102	2.58 [1.82;3.21]	2.14 [1.63;2.72]	0.04	1.75 [1.37;2.67]	2.20 [1.78;2.36]	0.247

CCRT: concurrent chemo-radiotherapy, Sqcc: squamous cell carcinoma, FIGO: International Federation of Gynecology and Obstetrics, RT: radiation therapy, PA: para-aortic region, TD: total dose, EQD2: equivalent dose in 2 Gy fractions, OT: overall treatment time, SCC0: pretreatment SCC antigen, SCC2: SCC antigen during CCRT, Cyfra0: pretreatment carcinoembryonic antigen 21-1, Cyfra2: carcinoembryonic antigen 21-1 during CCRT, Hb: hemoglobin (g/dL), PLT: platelet count $\times 10^{-3}$ (cells/µL), ANC: absolute neutrophil count (cells/µl), Mo: monocyte count (cells/µL), ALC: absolute lymphocyte count (cells/µl), CBC: compete bleed count (Hb, PLT, Mo, ANC, and ALC), CBC0: pretreatment CBC (Hb0, PLT0, Mo0, ANC0, and ALC0), Min CBC: minimum CBC during CCRT (min Hb, min PTL, min Mo, min ANC, and min ALC), CBC1: $\sqrt{\text{CBC0} \times \text{min CBC}}$ (Hb1, PLT1, Mo1, ANC1, and ALC1), CBC2: CBC second week during CCRT (Hb2, PLT2, Mo2, ANC2, and ALC2), CBC3: $\log\frac{\text{CBC2}}{\text{CBC0}}$ (Hb3, PLT3, Mo3, ANC3, and ALC3), NLR0: $\frac{\text{ANC0}}{\text{ALC0}}$, PLR0: $\frac{\text{PLT0}}{\text{ALC0}}$, LMR0: $\frac{\text{ALC0}}{\text{Mo0}}$, NLR1: $\frac{\text{ANC1}}{\text{ALC1}}$, PLT1: $\frac{\text{PLT1}}{\text{ALC1}}$, LMR1: $\frac{\text{ALC1}}{\text{Mo1}}$

Table S2. Cohort characterization of 42 cervical cancer patients, including exosomal RNA sequencing data (Cohort 2).

Factors	Mean ± standard deivation or n (%)	n
Age	51.5 ± 11.0	42
Pathology		42
Adenocarcinoma	7 (16.7%)	
Adeno-squamous cell carcinoma	1 (2.4%)	
Unknown carcinoma	1 (2.4%)	
Squamous cell carcinoma	33 (78.6%)	
FIGO stage		42
IB-IIIC1	24 (57.1%)	
IIIC2-IVB	18 (42.9%)	
Radiotherapy field		42
pelvis	31 (73.8%)	
pelvis and para-aortic region	11 (26.2%)	
Treatment time	54.9 ± 5.3	42
Total dose (EQD2)	75.9 ± 6.9	42
Follow up date	28.9 ± 15.2	42
Progression		42
No	31 (73.8%)	
Local progression	2 (4.8%)	
Distant metastasis	6 (14.3%)	
Local progression + distant metastasis	3 (7.1%)	
Follow up date	34.0 ± 13.2	42
Disease specific death		42
No	37 (88.1%)	
Yes	5 (11.9%)	

FIGO: International Federation of Gynecology and Obstetrics, EQD2: equivalent dose in 2 Gy fractions.

Table S3. Selection of early death patients in accordance with disease progression.

Sl. No.	Diagnosis date	Age	FIGO stage	Death	Time to death	Progression	Time to progression	2nd treatment	3rd treatment	4th treatment	Early death
1	2019	33	3C1	yes	5	LP+DM	3	CPB#2	CT#1		yes
2	2019	46	3C2	yes	5	LP+DM	3				yes
3	2019	37	1B	yes	6	DM	2	CPB#2	CT#1		yes
4	2018	61	4A	yes	25	DM	5	CPB#6			yes
5	2019	50	3C1	yes	34	LP+DM	6	CPB#8	CT#6	Pem#4	yes
6	2019	53	4B	loss『	7	DM	3	CPB#4			?
7	2020	44	3C2	no	30	DM	12	CPB#15	CT#6		?
8	2019	53	3C1	loss★	21	DM	20	RLLobectomy			no
9	2018	33	4B	no	49	DM	3	CPB#6			no
10	2019	46	3C2	no	45	LP	10	CPB#9			no
11	2019	57	3C1	no¥	43	LP	33	ICR			no

CPB: cisplatin, paclitaxel, and bevacizumab, CT: cisplatin and topotecan, Pem: pembrolizumab, RLLobectomy: right lower lobectomy, ICR: intracavitary brachytherapy 『refused further treatment despite partial response after four cycles of CPB, ★transferred to another hospital for further management after RLLobectomy owing to single lung metastasis, ¥performed ICR owing to cervical recurrence during follow-up after voluntary refusal of ICR on primary concurrent chemo-radiotherapy

In cohort 2 (42 patients), the occurrence of ED was ambiguous in two patients; among the remaining patient subset, propensity score matching was performed on five patients who experienced an ED and 10 patients who remained alive, in a 1:2 ratio.

Table S4. Comparison of hematological variables based on early death through propensity score matching

Early death	Yes (N=5)	No (N=10)	p
Age	45.40 ± 11.06	51.60 ± 13.65	0.396
Pathology			0.608
non-sqcc	2 (40.00%)	6 (60.00%)	
squamous cell carcinoma	3 (60.00%)	4 (40.00%)	
FIGO stage			0.56
1B-3C1	2 (40.00%)	2 (20.00%)	
3C2-4B	3 (60.00%)	8 (80.00%)	
Radiotherapy_field			1
pelvis	3 (60.00%)	7 (70.00%)	
pelvis and para-aortic region	2 (40.00%)	3 (30.00%)	
Total dose (EQD2)			0.282
-70.98	1 (20.00%)	0 (0.0%)	
-72.25	1 (20.00%)	1 (10.00%)	
-76.25	0 (0.0%)	4 (40.00%)	
-81.75	3 (60.00%)	5 (50.00%)	
duration	59.60 ± 2.61	54.10 ± 2.28	0.001
SCC0	6.60 [0.80;7.00]	1.90 [0.80;3.60]	0.383
SCC2	2.26 ± 1.69	2.02 ± 1.23	0.789
Cyfra0	10.20 [2.60;15.30]	1.80 [0.90;6.20]	0.147
**Cyfra2**	**2.20 [2.20;5.60]**	**1.65 [1.40;2.20]**	**0.067**
Hb0	12.40 ± 0.38	12.33 ± 2.19	0.924
min_Hb	9.28 ± 1.53	9.95 ± 1.48	0.428
Hb1	10.69 ± 0.86	11.06 ± 1.71	0.666
**Hb3**	**-0.20 ± 0.19**	**-0.02 ± 0.12**	**0.043**
PLT0	272.00 [239.00;281.00]	241.00 [212.00;311.00]	0.594
min_PLT	89.60 ± 49.89	111.50 ± 37.52	0.355
PLT1	147.63 ± 32.30	172.82 ± 55.97	0.373
PLT3	-0.37 ± 0.06	-0.41 ± 0.33	0.708
Mo0	540.20 [394.40;561.00]	423.50 [342.00;607.50]	0.462
min_Mo	167.42 ± 71.78	190.43 ± 91.22	0.632
Mo1	294.25 ± 76.92	290.36 ± 98.75	0.94
Mo3	-0.47 ± 0.35	-0.33 ± 0.40	0.506
**ANC0**	**5669.04 ± 2001.92**	**4026.93 ± 1173.06**	**0.064**
min_ANC	1581.00 ± 694.86	1263.31 ± 391.06	0.271
**ANC1**	**2910.69 ± 846.94**	**2202.60 ± 486.30**	**0.057**
ANC3	-0.44 ± 0.57	-0.23 ± 0.25	0.464
ALC0	1596.88 ± 160.85	1802.11 ± 413.87	0.311
Min ALC	135.32 ± 89.36	239.08 ± 136.96	0.151
ALC1	448.01 ± 153.82	632.68 ± 227.11	0.128
ALC3	-1.32 ± 0.19	-1.19 ± 0.47	0.569
**NLR0**	**3.63 ± 1.44**	**2.36 ± 1.03**	**0.071**
**NLR1**	**6.73 ± 1.64**	**3.82 ± 1.33**	**0.003**
PLR0	0.16 [0.14;0.17]	0.12 [0.10;0.18]	0.206

PLR1	0.35 [0.26;0.42]	0.25 [0.19;0.38]	0.31
LMR0	3.28 ± 1.32	4.18 ± 1.38	0.249
LMR1	1.58 ± 0.53	2.33 ± 1.01	0.146

CCRT: concurrent chemo-radiotherapy, Sqcc: squamous cell carcinoma, FIGO: International Federation of Gynecology and Obstetrics, PA: para-aortic region, EQD2: equivalent dose in 2 Gy fractions, SCC0: pretreatment SCC antigen, SCC2: SCC antigen during CCRT, Cyfra0: pretreatment Carcinoembryonic antigen 21-1, Cyfra2: Carcinoembryonic antigen 21-1 during CCRT, Hb: hemoglobin (g/dL), PLT: platelet $\times 10^{-3}$ (cells/μl), ANC: absolute neutrophil count (cells/μl), Mo: monocyte (cells/μl), ALC: absolute lymphocyte count (cells/μl), CBC: compete bleed count (Hb, PLT, Mo, ANC, and ALC), CBC0: pretreatment CBC (Hb0, PLT0, Mo0, ANC0, and ALC0), Min CBC: minimum CBC during CCRT (min Hb, min PTL, min Mo, min ANC, and min ALC), CBC1:$\sqrt{\text{CBC0} \times \text{min CBC}}$ (Hb1, PLT1, Mo1, ANC1, and ALC1), CBC2: CBC second week during CCRT (Hb2, PLT2, Mo2, ANC2, and ALC2), CBC3: $\log\frac{\text{CBC2}}{\text{CBC0}}$ (Hb3, PLT3, Mo3, ANC3, and ALC3), NLR0: $\frac{\text{ANC0}}{\text{ALC0}}$, PLR0: $\frac{\text{PLT0}}{\text{ALC0}}$, LMR0: $\frac{\text{ALC0}}{\text{Mo0}}$, NLR1: $\frac{\text{ANC1}}{\text{ALC1}}$, PLT1: $\frac{\text{PLT1}}{\text{ALC1}}$, LMR1: $\frac{\text{ALC1}}{\text{Mo1}}$

A substantial variation in Hb3 values was observed between two groups based on whether patients experienced early death (ED). This was attributable to the fact that one out of five patients who experienced ED in Cohort 2 possessed extremely low Hb3 values compared to those that did not.

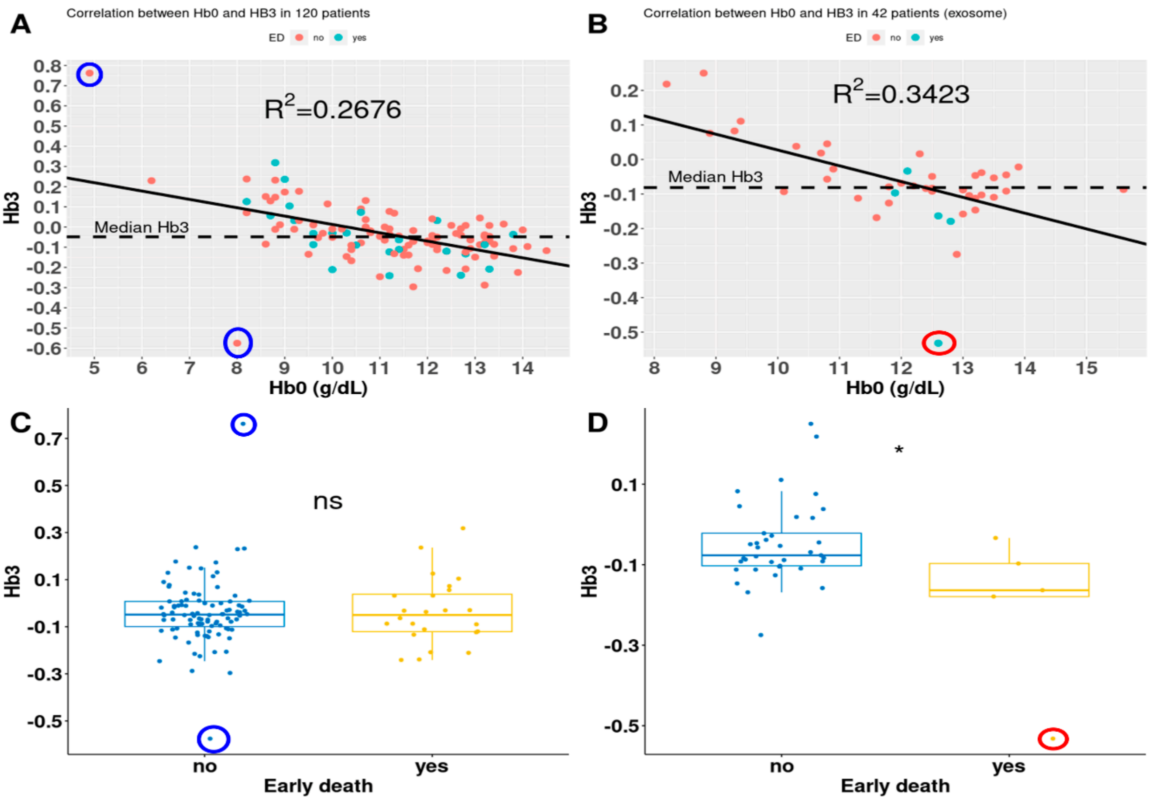

Figure S1. Graphical representation of the association between pretreatment hemoglobin (g/dL) (Hb0) and $\log\frac{\text{Hb2}}{\text{Hb0}}$ (Hb3) values in (A) cohorts 1 and (B) 2. Boxplots between two groups based on whether the patients suffered early death in (C) cohorts 1 and (D) 2. Hb2: second week during concurrent chemo-radiotherapy. ns: not significant, *: $p < 0.05$, circles symbolize outliers.

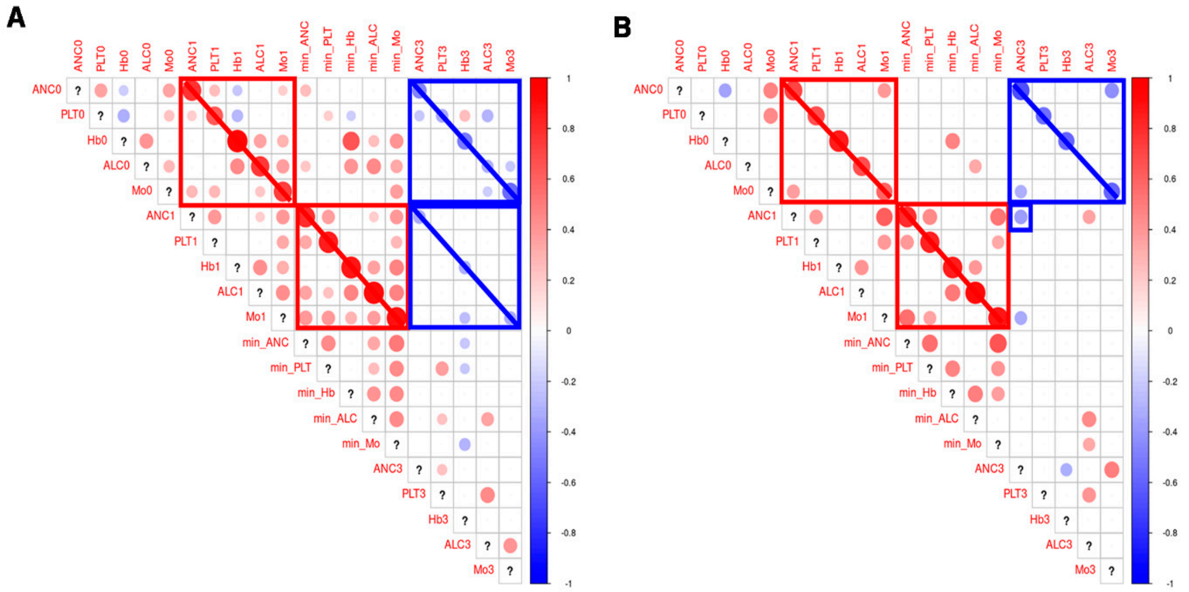

Figure S2. (A) Correlation matrix plot between CBC0, CBC1, CBC3, and min CBC of (A) 120 (cohort 1) and (B) 42 patients (cohort 2)

Hb: hemoglobin (g/dL), PLT: platelet count × 10^{-3} (cells/μL), ANC: absolute neutrophil count (cells/μl), Mo: monocyte count (cells/μl), ALC: absolute lymphocyte count (cells/μL), CBC: compete bleed count (Hb, PLT, Mo, ANC, and ALC), CBC0: pretreatment CBC (Hb0, PLT0, Mo0, ANC0, and ALC0), Min CBC: minimum CBC during CCRT (min Hb, min PTL, min Mo, min ANC, and min ALC), CBC1:$\sqrt{\mathrm{CBC0} \times \min \mathrm{CBC}}$ (Hb1, PLT1, Mo1, ANC1, and ALC1), CBC2: CBC during second week of CCRT (Hb2, PLT2, Mo2, ANC2, and ALC2), CBC3: $\log\frac{\mathrm{CBC2}}{\mathrm{CBC0}}$ (Hb3, PLT3, Mo3, ANC3, and ALC3)

Table S5. Number (%) of RNAs related to complete blood counts

Type	All CBC	CBC0	CBC1	CBC2	CBC3	Min CBC
Hb	7943 (48.3%)	4383 (54.4%)	2477 (42.5%)	1132 (18.3%)	3717 (53.9%)	1664 (39.4%)
ANC	4272 (26%)	775 (9.6%)	814 (14%)	1908 (30.8%)	1678 (24.3%)	685 (16.2%)
ALC	3605 (21.9%)	1669 (20.6%)	1148 (19.7%)	853 (13.8%)	914 (13.3%)	768 (18.2%)
Mo	4656 (28.3%)	2120 (26.3%)	899 (15.4%)	1178 (19%)	802 (11.6%)	953 (22.6%)
PLT	4653 (29.3%)	1112 (23.8%)	1426 (24.5%)	1863 (30%)	754 (10.9%)	1261 (29.9%)

CCRT: concurrent chemo-radiotherapy Hb: hemoglobin (g/dL), PLT: platelet × 10^{-3} (cells/μl), ANC: absolute neutrophil count (cells/μl), Mo: monocyte (cells/μl), ALC: absolute lymphocyte count (cells/μl), CBC: compete bleed count (Hb, PLT, Mo, ANC, and ALC), CBC0: pretreatment CBC, Min CBC: minimum CBC during CCRT, CBC1:$\sqrt{\mathrm{CBC0} \times \min \mathrm{CBC}}$, CBC2: CBC second week during CCRT, CBC3: $\log\frac{\mathrm{CBC2}}{\mathrm{CBC0}}$

All CBC: the number of RNAs associated with at least one of CBC0, CBC1, CBC2, CBC3, or min CBC

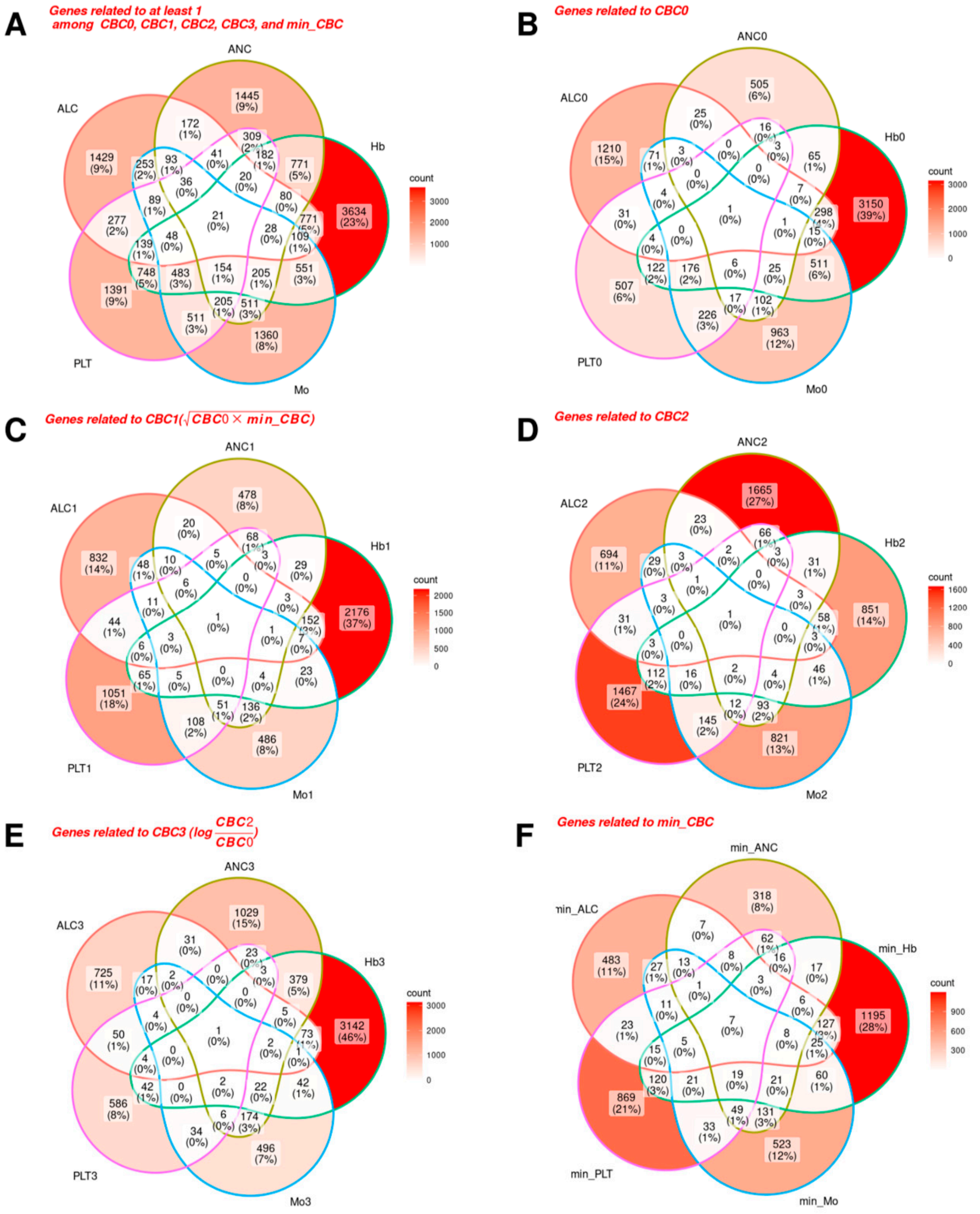

Figure S3. Venn diagrams illustrating the relationships of RNAs relevant to various measures, including (A) all CBC, (B) CBC0, (C) CBC1, (D) CBC2, (E) CBC3, and (F) min CBC.

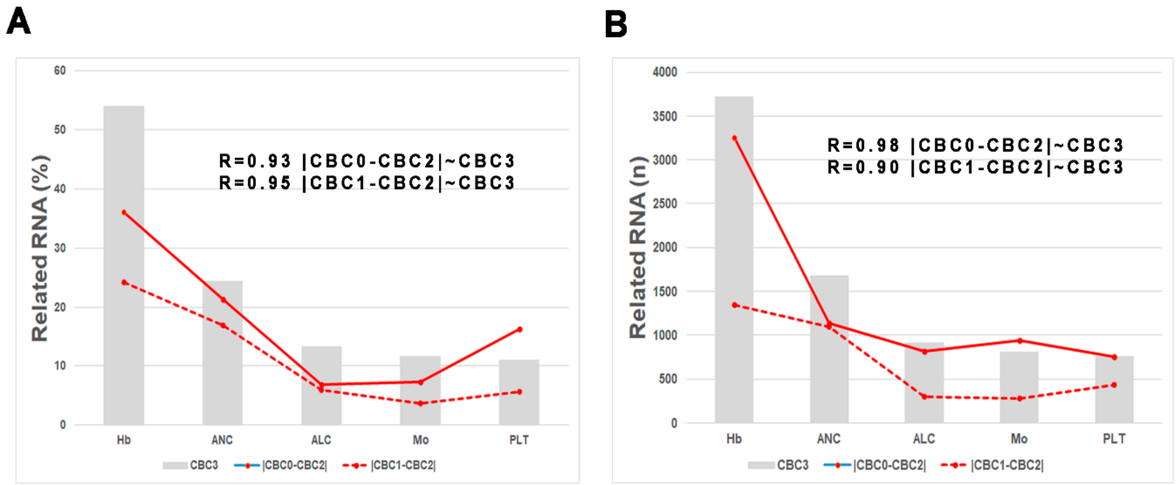

Figure S4. (A) Percentage and (B) number of RNAs relevant to CBC3, |CBC0-CBC2|, and |CBC1-CBC2| according to hematological parameters.

R: Pearson's correlation coefficient, |CBC0-CBC2|: the absolute difference between the number/percentage of RNAs associated with CBC0 and those with CBC2, |CBC1-CBC2|: the absolute difference between the number/percentage of RNAs associated with CBC1 and those with CBC2

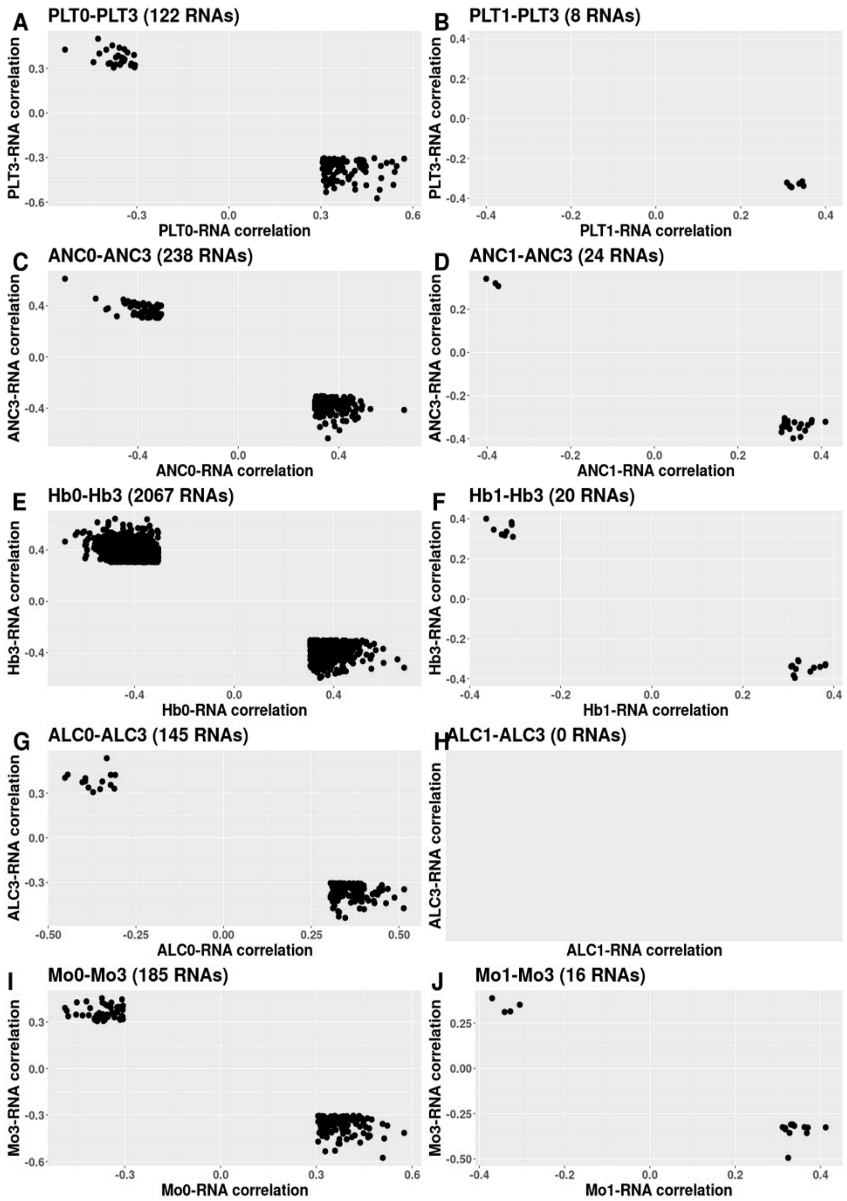

Figure S5. Scatter plots between CBC0-RNA and CBC3-RNA and between CBC1-RNA and CBC3-RNA correlation coefficients.

Table S6. Clinical information in descending order of miR-574-3p-LINC01003-ACOT9 (log_2FC) values > zero.

Sl. No.	Diagnosis date	Age	Pathology	FIGO stage	Early death	Time to death	Progression	Time to progression	miR-574-3p	LINC01003	*ACOT9*	miR-574-3p-LINC01003-*ACOT9* (log_2FC)
1	2019	37	adeno, mucinous	1B	yes	6	DM	2	0.029505	-5.9484	0.146573	5.831331
2	2019	33	adeno, gastric	3C1	yes	5	LP+DM	2	0.905798	-3.27729	-1.27091	5.454002
3	2019	46	sqcc	3C2	yes	5	LP+DM	3	0.964931	-1.93587	-1.38629	4.287088
4	2019	50	adeno-sqcc	3C1	yes	34	LP+DM	6	0.159406	-3.41579	0.060288	3.514905
5	2018	61	sqcc	4A	yes	25	DM	5	1.415483	0.025887	-1.879	3.268595
6	2019	53	adeno-sqcc	4B	loss	7	DM	3	-0.04338	-0.38931	-2.37895	2.724888
7	2020	44	adeno, gastric	3C2	no	30	DM	12	0.189702	-0.41053	-1.8549	2.455125
8	2019	52	sqcc	3C1	no	40	no	40	0.426518	-2.92084	0.994822	2.352531
9	2019	37	sqcc	3C1	no	40	no	40	-0.12042	-1.9207	0.190166	1.610119
10	2018	39	sqcc	3C1	no	47	no	47	-0.89175	-1.48783	-0.72384	1.319917
11	2019	63	sqcc	1B	no	38	no	38	0.421173	0.167139	-0.64469	0.89872
12	2019	39	sqcc	3C1	no	44	no	35	0.303904	-0.60316	0.454039	0.453027
13	2018	33	sqcc	4B	no	49	DM	3	0.21782	-0.94736	0.872771	0.292411
14	2020	40	sqcc	3C1	no	33	no	33	-0.17821	-0.10284	-0.11716	0.041788

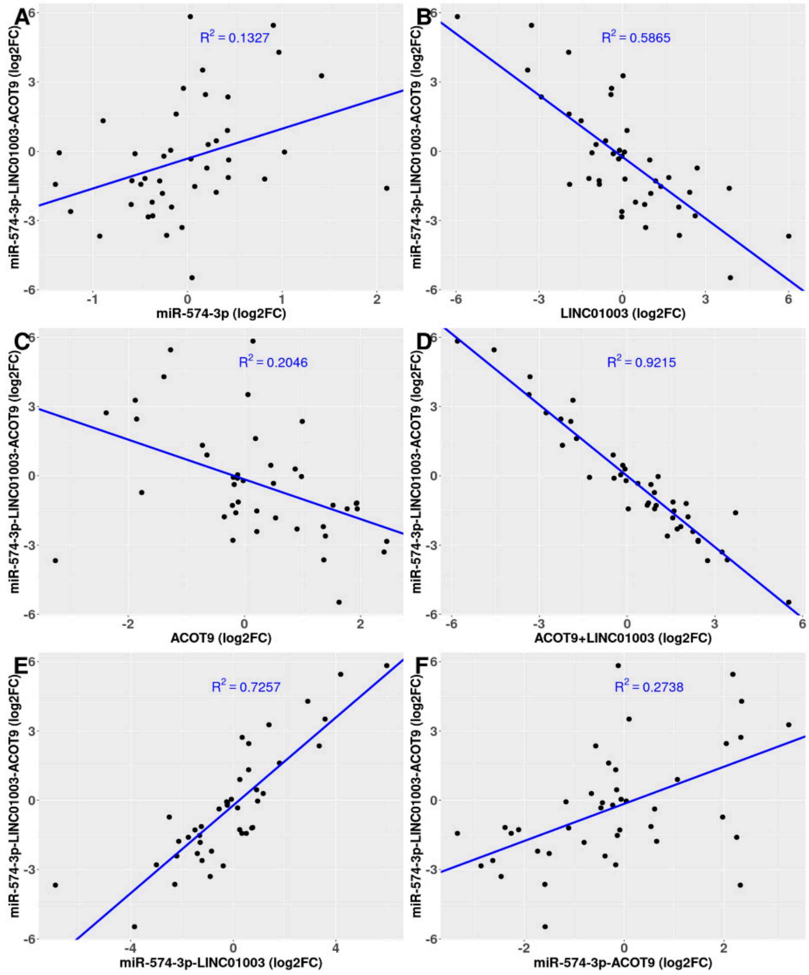

Figure S6. Linear regressions between the $\log_2$ fold change ($\log_2$ FC) values of miR-574-3p-LINC01003-*ACOT9* and their constituent elements.
