# Supplementary material for: Post-Radiotherapy Exosomal Non-Coding RNA and Hemograms for Early Death Prediction in Patients with Cervical Cancer
Source: Int J Mol Sci. 2023 Dec 21;25(1):126. doi: 10.3390/ijms25010126 (PMC10778718; doi:10.3390/ijms25010126)
Supplement: Supplementary file 1 [file ijms-25-00126-s001.zip › supplement_methods-english.pdf]

Patients

The pathological findings of all the patients were confirmed via cervical biopsy. Local staging, regional lymph node (LN), and distant metastasis were evaluated using magnetic resonance imaging (MRI), computed tomography (CT), or positron emission tomography–CT. Suspicious bladder or rectal invasion was confirmed via cystoscopy or sigmoidoscopy. External beam radiotherapy (RT) (EBRT) was delivered using 10–15 MV photons to the pelvis with or without para-aortic LN. The pelvic RT dose was 45 Gy, delivered in 25 fractions, and the LN lesions were boosted up to 55–60 Gy. The RT dose of the metastatic sites in 15 of the patients with stage IVB was 30–55 Gy, delivered in 10–25 factions. One patient with a single lesion in the left lung was treated with stereotactic body RT using 48 Gy in four fractions. The 460 patients, except 13 who switched to EBRT, underwent high-dose rate intracavitary brachytherapy (Iridium-192; Microselectron, Nucletron, Veenendaal, Netherlands, or GammaMedplus iX, Varian, Palo Alto, CA, USA). Weekly cisplatin (30–70 mg/m^2) was administered in four to six cycles during RT in all the patients. The patients were followed-up every 1–3 months after treatment completion. Primary cervical tumors, regional LN, and distant metastases were evaluated through pelvic examination, Pap smear tests, tumor markers, MRI, and CT.

Variables

Information about the age at diagnosis, pathology, 2018 International Federation of Gynecology and Obstetrics stage, total dose (TD), overall treatment time (OT), RT field, tumor markers such as pretreatment squamous cell carcinoma antigen (SCC-Ag), pretreatment carcinoembryonic cytokeratin 19 fragment antigen 21-1 (Cyfra), SCC-Ag during concurrent chemoradiotherapy (CCRT), and Cyfra during CCRT was collected from all the patients. TD was the sum of the EBRT and brachytherapy dose administered to the central lesion (equivalent dose in 2 Gy fractions). The follow-up time was measured as the duration between the end of CCRT and the occurrence of the event (progression or disease-specific death).

Blood sampling timeline and summary of the treatment process

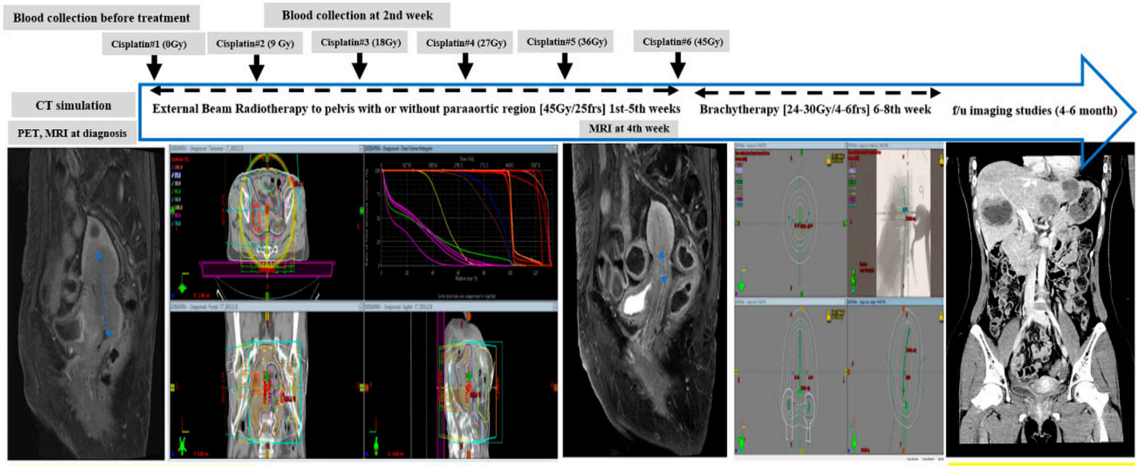

Complete blood counts of all patients who had undergone weekly cisplatin-based CCRT were measured before the administration of cisplatin using Beckmann Coulter analyzer DxH 900. The blood sampling timeline for the transcriptomic analysis within exosomes isolated from the plasma of 42 cervical patients treated with primary CCRT is also presented (Cohort 2).

Plasma exosomal ribonucleic acid (RNA) sequencing process

Plasma preparation and storage

Blood was collected in an ethylenediaminetetraacetic acid tube (purple stopper), stored at 4°C, and centrifuged within 2 h at 13,000 rpm and 4°C for 10 min. The uppermost yellow layer of the centrifuged blood was divided into 300 μl aliquots in sterilized cryotubes; then, the tube cap was labeled according to the assigned blood resource number. For long-term plasma storage, the conditions were pp tube, 0.5–2 ml, and −85 to −60°C. The storage period of the samples before being used for analysis ranged from 3 to 12 months.

Small RNA library construction and sequencing

Exosomes were isolated from human plasma by mixing the plasma with Exo2D RNA solution (Exosomeplus). We chose a clinically applicable method that demonstrated stability and consistently yielded a high quantity of exosomes over time. This method involved the extraction of exosomes using an aqueous two-phase system, which was tailored to the surface characteristics of exosomes (Shin, H., Han, C., Labuz, J. *et al.* High-yield isolation of extracellular vesicles using aqueous two-phase system. *Sci Rep* **5**, 13103 (2015).

https://doi.org/10.1038/srep13103). This exosome isolation kit guaranteed a particle recovery rate of more than 90%. Particle recovery means that after separation (and concentration) using the Exo2D kit, the exosomes from which impurities have been removed are preserved by more than 90% compared to before separation. The detailed isolation process was performed according to the manufacturer's instructions, attached separately. RNA from plasma-derived exosomes was extracted using the miRNeasy Serum/Plasma Kit (Qiagen, Valencia, CA) according to the manufacturer's instructions, attached separately. The RNA concentration was calculated using Quant-IT RiboGreen (Invitrogen). The RNA size was confirmed using an Agilent RNA 6000 Pico Kit, a Small RNA Kit, and an Agilent 2100 Bioanalyzer (Agilent Technologies, Böblingen, Germany). A total of 10 ng of RNA was isolated from each sample and used to construct sequencing libraries using the Simple Modular Architecture Research Tool (SMART)er small RNA (smRNA)-Seq Kit from Illumina following the manufacturer's protocol. In this method, input RNA is first polyadenylated to provide a priming sequence for an oligo(dT) primer. Copy deoxyribonucleic acid (cDNA) synthesis is primed by the 3' smRNA dT Primer, which incorporates an adapter sequence at the 5'end of each RNA template. It adds non-templated nucleotides that are bound by the SMRT smRNA Oligo-enhanced with locked nucleic acid technology for greater sensitivity. In the template-switching step, PrimeScript RT uses the SMART smRNA Oligo as a template for the addition of a second adapter sequence to the 3'end of each first-strand cDNA molecule. In the next step, full-length Illumina adapters (including index sequences for sample multiplexing) are added during polymerase chain reaction (PCR) amplification. The forward PCR primer binds to the sequence added by the SMART smRNA Oligo, while the reverse PCR primer binds to the sequence added by the 3′ smRNA dT Primer. The amplified libraries were purified from 6% Novex tetrabromoethane–polyacrylamide gel electrophoresis gels (Thermo Fisher, MA) to excise bands with a size of approximately 138 bp (over than 18 bp of cDNA plus 120 bp of adaptors). The resulting library of cDNA molecules included the sequences required for clustering on an Illumina flow cell. The libraries were gel purified and validated by checking their size, purity, and concentration on an Agilent Bioanalyzer. The libraries were quantified using quantitative PCR (qPCR) according to the qPCR Quantification Protocol Guide (KAPA Library Quantification kits for Illumina Sequencing platforms) and qualified using TapeStation D1000 ScreenTape (Agilent Technologies, Waldbronn, Germany). The libraries were then pooled in equimolar amounts and sequenced using an Illumina HiSeq 2500 (Illumina, San Diego, USA) instrument to generate 51 base reads. Image decomposition and quality value calculations were performed using the modules of the Illumina pipeline.

Adapter trimming

The raw sequencing reads of the smRNAs from the different experimental samples were pre-processed and analyzed using miRDeep2. Adapter trimming was performed using the cutadapt program to eliminate the adapter sequences that existed in the reads that were attached to the micro-RNA (miRNA) during the smRNA library construction process. The first 3 nucleotides of all the reads were trimmed to remove extra bases inserted during the SMART template-switching activity process. The adapter sequence and other sequences at the 3′ end of the adapter were also removed. If a read matched more than at least first five base pairs of the 3′ adapter sequence, it was regarded as an adapter sequence and trimmed from the read. Trimmed reads should have a minimum of 18 bp in order to be considered reliable for analysis. The remaining reads whose sequences did not match the adapter sequence were classified as non-adapter reads. In this analysis, the trimmed and non-adapter reads were combined and regarded as processed reads for downstream analysis.

Clustering

To minimize sequence uniqueness and computational intensity, processed adapter sequence reads were gathered to form a cluster. This cluster contained reads that showed a 100% match with the sequence identity and read length, and it was assigned a temporary cluster ID along with the number of reads it held.

Ribosomal RNA (rRNA) filtering

In order to eliminate the effects of large amounts of rRNA, the reads were aligned to the 45S pre-rRNA and mitochondrial rRNA sequences of *Homo sapiens* and matched.

Messenger RNA (mRNA) expression profiling

The reference gene annotation for *Homo sapiens* (GRCh38; release 109.20190607) was retrieved from the National Center for Biotechnology Information. Since the produced read contained not only smRNA but also mRNA, mRNA expression profiling was performed using RSEM (v1.3.1) with options (--estimate-rspd --seed-length 15 --strandedness forward).

Identification of known miRNA reads

Sequence alignment and detection of known and novel miRNAs were performed using the miRDeep2 software algorithm. Prior to performing sequence alignment, the *Homo sapiens* reference genome was indexed using Bowtie (1.1.2) for aligning sequencing reads to the reference sequences. Those reads were then aligned with the

mature and precursor miRNA sequences of *Homo sapiens* obtained from miRBase v21. The miRDeep2 algorithm is based on the miRNA biogenesis model; it aligns the reads to potential hairpin structures in a manner consistent with Dicer processing and assigns scores that represent the probability that hairpins are true miRNA precursors. In addition to detecting known and novel miRNAs, miRDeep2 estimates their abundance.

Proportion of miRNA and other RNA types

Uniquely clustered reads were then sequentially aligned to the reference genome using miRBase v21 and non-coding RNA database RNAcentral release 10.0 to identify known miRNAs and other types of RNA.
